# The identification of primary care consultation visits for otitis media: development of a software algorithm screening tool

**DOI:** 10.1017/S1463423625100674

**Published:** 2026-01-05

**Authors:** Cameron Charles Grant, Marisa van Arragon, Ellen Waymouth, Alicia Stanley, Mapui Tangi, Eamon Ellwood, Carol Chelimo

**Affiliations:** 1 Department of Paediatrics: Child & Youth Health, University of Auckland, Auckland, New Zealand; 2 General Paediatrics, Starship Children’s Hospital, Te Whatu Ora - Health New Zealand Te Toka Tumai Auckland, Auckland, New Zealand; 3 Department of Critical Care Medicine Research, Auckland City Hospital, Te Whatu Ora – Health New Zealand Te Toka Tumai Auckland, Auckland, New Zealand; 4 Clinical Nurse Specialist, Kidz First Children’s Hospital, Te Whatu Ora – Health New Zealand Counties Manukau, Auckland, New Zealand

**Keywords:** electronic health records, primary health care, otitis media

## Abstract

**Background:**

Identifying diagnoses from noncoded healthcare visit records presents logistical challenges when large number of records are screened. This study aimed to develop a screening process to identify otitis media (OM) diagnoses in free-text primary care visit records.

**Methods:**

The free-text primary care records of 200 children aged 0 to 4 years were reviewed independently by three clinicians to determine whether OM was a diagnosis considered during each visit. Terms (abbreviations, words, and phrases) identifying visits where OM was considered or excluded were documented. These terms were used to design a software algorithm subsequently used to detect OM diagnosis within these primary care records. The diagnostic performance of the software algorithm was determined against the gold standard clinicians’ review and described using sensitivity, specificity, predictive values (PVs), and likelihood ratios (LRs) with 95% confidence intervals (CIs).

**Results:**

The 200 children had 10,034 primary care visits. Clinician review identified 917 (9%) visits where OM was considered, and 9117 (91%) visits where OM was excluded. The software algorithm identified 801/917 visits where OM was considered and 8705/9117 visits where OM was excluded. The algorithm sensitivity was 87% (95% CI 85–89), specificity 96% (95% CI 95–96), positive PV 66% (95% CI 63–69), negative PV 99% (95% CI 98–99), positive LR 19.33 (95% CI 17.54–21.31), and negative LR 0.13 (95% CI 0.11–0.16).

**Conclusion:**

Software algorithms can assist in screening healthcare visit records. When combined with clinician review, they enable accurate identification of OM visits from non-coded records.

## Background

Parental report of otitis media (OM) is an outcome measure used when investigating the relationship of early childhood ear infections with subsequent hearing impairment and poorer psychosocial health (Yiengprugsawan *et al.*, 2013; Hogan *et al.*, 2014). However, parental report of OM is not a robust measure of OM primary care visits. In an audit of primary care illness visits for children <18 months old living in Auckland, New Zealand (NZ), parental report underestimated the number of primary care doctor visits for acute respiratory illnesses (Grant *et al.*, 2015). Here, parents reported approximately one-in-three visits identified by clinician review of the primary care records (Grant *et al.*, 2015).

When primary care records are uncoded, clinician review of the free-text description of each primary care visit is possible but impractical for larger studies. As an alternative, software algorithms have been developed to scan and detect diagnoses in free-text records (Aronson and Lang, 2010). This computer software approach involves scanning free-text records for abbreviations, words and phrases related to the illness of interest.

However, relying solely upon a software algorithm to identify OM healthcare visits introduces the potential for errors. A significant problem is created by ambiguous abbreviations, commonly used in medical records (Sheppard *et al.*, 2008; Collard and Royal, 2015; Awan *et al.*, 2016). Abbreviations can have different meanings in different clinical specialties and contexts. For example, ‘ROM’ is an abbreviation used for both ‘right otitis media’ and ‘range of movement’.

Another error that can occur with software algorithms is that they ignore the context of a clinical record entry. For example, the phrase ‘brother has otitis media’ could mistakenly be identified by a software algorithm as a visit for OM in the index child.

This study aimed to develop a screening process to identify OM diagnoses in free-text primary care consultation visits. We intended to create a software algorithm with high specificity to rule-out primary care visits that were not for OM. Keeping false positives to a minimum diminishes the subsequent clinician record review workload required when conducting primary care record-based research about OM.

## Methods

This study was completed using the *Growing Up in New Zealand* child cohort study data (Morton *et al.*, 2013). This cohort included 6853 children born to women residing within a geographically defined region of NZ who had an estimated delivery date between April 25^th^, 2009 and March 25^th^, 2010 (Morton *et al.*, 2013). The NZ Ministry of Health Northern Y Regional Ethics Committee granted ethical approval (NTY/08/06/055) for the study. When the children were 54 months old, the study obtained written informed consent from the child’s caregiver to access the child’s primary care records. In NZ, primary care records are those created by General Practitioners (family doctors) working from primary care practices situated outside of the hospital setting.

The primary care records for a convenience sample of 200 children up to age 4 years from the *Growing Up in New Zealand* cohort were reviewed (Morton *et al.*, 2013). These records contained the free-text description of the child’s visit recorded by the various General Practitioners working in 20 different primary care practices. Inclusion of free text records created by different General Practitioners, working in several practices, allowed us to consider individual variability in free text record documentation.

A team of three clinicians, comprising a paediatrician (CG) and two research nurses (MvA, EW), reviewed these records. First, clinicians independently determined whether OM was a diagnosis considered during the consultation and documented the abbreviations, words, and phrases encountered during this process.

At the second stage, clinicians met and reviewed each consultation record to confirm agreement about the OM diagnoses identified. Where opinions differed, the consultation record was reviewed alongside the list of terms documented by clinicians to classify OM as present or absent, with consensus reached through this process.

During the third stage, the study data manager used the list of terms documented by the clinicians to design the software algorithm and screen the same set of clinician-reviewed primary care records (supplementary material). The development of the screening process for identification of OM diagnoses within primary care records involved comparing the results of the clinician and software algorithm screening methods. The clinicians and data manager met to discuss records where the results of the clinician and computer algorithm review differed, in particular, the false positive records identified as a visit where OM was present by the software algorithm but not the clinician review. The list of terms was also reviewed and corrected during this process.

During the fourth stage, the software algorithm was re-run based upon the updated list of terms. The performance of the software algorithm was assessed, with the clinician review as the gold standard. The results were described using sensitivity, specificity, positive and negative predictive values (PVs), and likelihood ratios (LRs) with 95% confidence intervals (CIs) (Chu, 2002). We sought to develop a software algorithm with high specificity and high negative predictive value as we wanted to identify all consultations where OM may have been considered (Akobeng, 2007), and thus required clinician review.

## Results

The 200 children had a total of 10,034 primary care visits (Table 1). Clinician review determined that OM was considered a diagnosis in 917 (9%) primary care visit records. In 9117 (91%) primary care consultation visits, OM was an excluded diagnosis.

**Table 1.** Summary of primary care visits and primary care otitis media visits for the 200 children from the 20 primary care practices included in the pilot study

Practice	Number of children per practice(*n* = 200)	Otitis media visits per practice by clinician file reading	Otitis media visits per child per practice by clinician file reading	Otitis media visits per practice by computer file reading	Otitis media visits per child per practice by computer file reading	Total primary care visits per practice by the children included in the pilot study
1	1	0	0.00	0	0.00	15
2	5	65	13.00	100	20.00	627
3	2	6	3.00	7	3.50	83
4	1	6	6.00	9	9.00	40
5	16	60	3.75	85	5.31	460
6	1	1	1.00	1	1.00	20
7	11	26	2.36	58	5.27	583
8	2	2	1.00	2	1.00	11
9	19	60	3.16	76	4.00	706
10	7	21	3.00	20	2.86	156
11	44	172	3.89	248	5.64	2426
12	3	35	11.67	30	10.00	295
13	4	12	3.00	22	5.50	304
14	13	86	6.62	107	8.23	660
15	7	23	3.29	32	4.57	212
16	1	3	3.00	7	7.00	122
17	32	173	5.41	216	6.75	2370
18	27	149	5.52	172	6.37	752
19	2	10	5.00	12	6.00	46
20	2	7	3.50	9	4.50	146
						10034

Table 2 contains the list of terms documented by clinicians classifying OM as a diagnosis recorded in the primary care records. When any of the terms from Table 2 were combined with words from Table 3, OM was excluded. For example, if a term referring to the tympanic membrane, i.e., ‘T/M’, ‘t/m’, ‘tm’ or ‘TM’ was combined with the word ‘normal’ in Table 3, OM was excluded as a reason for the primary care visit.

**Table 2.** Search terms which identified a primary care visit where otitis media was a potential reason for the primary care visit

‘AOM’ ‘aom’ ‘ASOM’ ‘BOM’ ‘drum’ ‘LAOM’ ‘O M’ ‘O Med’ ‘OM’ ‘om’ ‘OME’ ‘ome’ ‘RAOM’ ‘red ear’ ‘ROM’ ‘tm’ ‘TM’ ‘tms’ ‘AOM’ ‘aom’ ‘BOM’ ‘BOME’ ‘bulging’	‘bulging TM’ ‘Cochlear’ ‘cochlear’ ‘discharging ear’ ‘drum’ ‘Drum’ ‘Drums’ ‘drums bulging’ ‘ear red’ ‘ear - red’ ‘ear discharge’ ‘ear dull’ ‘ear infected’ ‘ear infection’ ‘ear inflamed’ ‘ear inflammed’ ‘ear pus’ ‘ear red’ ‘eardrums red’ ‘ears - dull’ ‘ears – inflamed’ ‘ears red’ ‘ears – red’	‘ears dull’ ‘ears infected’ ‘ears inflamed’ ‘ears inflammed’ ‘ears red’ ‘effusion’ ‘glue ear’ ‘gromet’ ‘grommet’ ‘grommets’ ‘Grommets’ ‘inflame ear’ ‘inflamed ear’ ‘inflammed ear’ ‘injected’ ‘LOM’ ‘LOME’ ‘O M’ ‘O media’ ‘om/’ ‘Ot Media’ ‘Ot. Media’ ‘otitis’	‘Otitis’ ‘otitis media’ ‘Otitis Media’ ‘Otitis media’ ‘red drum’ ‘red eardrums’ ‘ROM’ ‘ROME’ ‘T/M’ ‘t/m’ ‘tm’ ‘TM’ ‘TM bulging’ ‘tm dull’ ‘TM infected’ ‘tm infected’ ‘TM inflammed’ ‘TM red’ ‘tm red’ ‘tms’ ‘tms bulging’ ‘tms dull’

**Table 3.** Words which in combination with a search term* from Table 3 identified a primary care visit where otitis media was excluded as a potential reason for the primary care visit

[searchterm] and ‘nad’ [searchterm] and ‘externa’ [searchterm] and ‘NAD’ [searchterm] and ‘ok’ [searchterm] and ‘OK’ [searchterm] and ‘not’ [searchterm] and ‘nad’ [searchterm] and ‘NAD’ [searchterm] and ‘ok’ [searchterm] and ‘OK’ [searchterm] and ‘not’ [searchterm] and ‘s not’ [searchterm] and ‘n’ [searchterm] and ‘n’ [searchterm] and ‘s n’ [searchterm] and ‘s OK’ [searchterm] and ‘s normal’ [searchterm] and ‘s nad’ [searchterm] and ‘s ok’ [searchterm] and ‘s clear’ [searchterm] and ‘normal’	[searchterm] and ‘prazole’ [searchterm] and ‘eprazole’ [searchterm] and ‘an’ [searchterm] and ‘normal’ [searchterm] and ‘healthy’ [searchterm] and ‘clear’ [searchterm] and ‘clear’ [searchterm] and ‘L’ [searchterm] and ‘pharynx’ ‘no’ and [searchterm] ‘no’ and [searchterm] ‘f’ and [searchterm] ‘full’ and [searchterm] ‘full’ and [searchterm] ‘good’ and [searchterm] ‘active’ and [searchterm] [searchterm] and ‘office’ ‘Growing up in NZ study’ and [searchterm] ‘Growing up in NZ study,’ and [searchterm] ‘Growing up in New Zealand Study’ and [searchterm] ‘NIR’ and [searchterm]

* When one of the search terms listed in Table 3 is found in the primary care visit text, it is then compared to all the exclusion terms. If the search term + exclusion term combination matches the visit text for any of the exclusion terms the search term is excluded from the results.

The software algorithm correctly identified 801 of the 917 primary care visits where clinician review determined that OM was considered a diagnosis and 8705 of the 9117 primary care visits where clinician review determined that OM was an excluded diagnosis.

In comparison with the clinician review, the software algorithm had the following diagnostic test properties (95% CI): sensitivity 87% (85–89%); specificity 96% (95–96%); positive predictive value 66% (63–69%); negative predictive value 99% (98–99%); positive LR 19.33 (17.54–21.31); and negative LR 0.13 (0.11–0.16) (Table 4).

**Table 4.** Diagnostic performance of the electronic record review compared with the clinician review as the reference for identifying primary care visits where otitis media was a potential reason for the primary care visit

		Otitis media a potential reason for the primary care visit as determined by clinician audit		Total
		Yes	No	
Otitis media a potential reason for the primary care visit as determined by electronic review	Yes	801	412	1213
	No	116	8705	8821
Total		917	9117	10034 primary care visits made by the 200 children

Diagnostic test parameter (95% confidence intervals). Sensitivity = 87.35% (85.02% to 89.43%) Specificity = 95.48% (95.03% to 95.9%) Negative predictive value = 98.68% (98.42% to 98.91%) Positive predictive value = 66.03% (63.29% to 68.7%).

## Discussion

### Statement of principal findings

This study describes the process used to create a software algorithm to search free-text primary care visit records to identify primary care visits where OM was one of the diagnoses made. The intention was to create an algorithm with high specificity to rule out primary care visits that were not for OM. The negative predictive value of the software algorithm developed was 99% (95% CI 98–99%). The positive LR was 19.33 and negative LR was 0.13. The positive and negative LRs derived from this algorithm are of a magnitude that result in large increases in the post-test probability for the positive LR (>10) and moderate increases in the post-test probability for the negative LR (0.10–0.20)(Grimes and Schulz, 2005).

The software algorithm identified a subset of primary care records that required clinician review to determine the presence or absence of OM. The clinician review determined that 66% of this subset of primary care visits were due to OM. Thus, the software algorithm reduced the number of primary care records visits requiring clinician review by 88%, from 10034 to 1213 visits. By reducing the number of healthcare visit records requiring clinician review, this greatly reduced the cost in terms of time and budget to extract data from free text primary care records and create variables that precisely and accurately define OM primary care visits.

### Study strengths and weaknesses

The use of primary care records that are uncoded, whilst requiring the development of data extraction processes such as described here, has some advantages. Coding of primary care visits varies in its completeness. Respiratory illness visits in preschool aged children is a visit type previously shown to have a relatively high rate of incomplete coding (Hansell *et al.*, 1999). More recent studies reporting primary care practice audit data document that variability in coding completeness persists (Molony *et al.*, 2016). Barriers to complete coding include the limitations of the coding systems, variability in coding skills and motivation, time pressures preventing the structured recording of data during consultations, and the priority given to coding within organizations (de Lusignan, 2005).

The study was completed using General Practitioner primary care records only. It may not be applicable to other settings where primary care is delivered, such as hospital emergency departments. This study records of primary care visits made during the preschool years for children born in 2009–10 (Morton *et al.*, 2013). Free-text descriptors can change over time and are likely to vary between countries. It is important that the development of any algorithm includes a piloting process, such as that described here, that is completed within the primary care record collection to which the algorithm is to be applied.

The algorithm was developed so it could identify when OM was specifically excluded. The algorithm was intentionally developed to be used in combination with a clinician review and hence, we cannot recommend its use without being combined with clinician review.

The list of search terms we developed included abbreviations and acronyms. Abbreviations and acronyms are commonly used in medical records (Awan *et al.*, 2016). Our study showed the numerous abbreviations used for the diagnosis of ‘otitis media’ (Table 2). Patterns of abbreviation and acronym use vary between countries and in different clinical settings (Sheppard *et al.*, 2008, Collard and Royal, 2015). Hence, the process described here would need refinement before being used in different countries and different clinical settings.

Medical record search methods which only include a software algorithm review, without clinician review, miss events where short abbreviations are used in the text (Shah *et al.*, 2012; MacRae *et al.*, 2015a). Examples cited in the literature of abbreviations not identified correctly include MI for myocardial infarction and ‘regurg’ for regurgitation (Shah *et al.*, 2012).

Without clinician review, the issue of how acronyms, abbreviations and misspellings are measured or addressed is not possible to define (Wu *et al.*, 2013; MacRae *et al.*, 2015a). With meanings of and patterns of use of abbreviations varying between clinical settings, it would be necessary to repeat the process described in this study for identifying OM diagnoses in other settings. Whilst algorithms can be developed which expand abbreviations into more complete text statements, this is not possible when the abbreviation has multiple and different meanings (Chapman *et al.*, 2005). Misspellings occur frequently in medical notes created using a keyboard, due to both spelling proficiency and keyboard skills, and also differences in personal practice behaviours related to checking for and correcting misspellings in medical records (MacRae *et al.*, 2015b).

### Strengths and weaknesses in relation to other studies

Clinician review and classification of clinical records is acknowledged as time consuming and prone to error. However, it remains a necessary component for extracting data from free-text healthcare visit records. Contemporary systematic reviews of the secondary use of clinical data contained in electronic medical records for research purposes highlight the high frequency of data quality issues and data pre-processing challenges (Edmondson and Reimer, 2020; Lewis *et al.*, 2023).

In this study we had three clinicians classifying visits independently and reaching consensus when initial independent classifications of individual visits differed. Having multiple clinicians involved reduces the bias that can occur if only one clinician is classifying visits, as has been used in another recent study which sought to develop an algorithm for identifying influenza-like illness visits from uncoded General Practitioner records (MacRae *et al.*, 2015b).

In countries, such as NZ, where acute respiratory infection primary care presentations are not consistently coded, reliance solely on a computer programme has insufficient sensitivity to identify visits for childhood respiratory illness presentations. For example, in a study that used 1200 primary care records of children aged 0 to 18 years attending 10 different primary care practices in Wellington, NZ, a software algorithm developed specifically to read primary care records had a sensitivity of 58% and specificity of 99% for identifying primary care OM visits (MacRae *et al.*, 2015a). The computer programme enabled non-OM visits to be excluded, thus significantly reducing the number of records requiring clinician review. However, a clinician review of those records identified by the computer programme as OM visits is still required.

In addition to the presence of acronyms, abbreviations and misspellings, the lack of well-formed grammar or structure in these free-text primary care records limit the capacity to use computer software to identify phrases which reliably correspond to clinical diagnoses (MacRae *et al.*, 2015b).

### Meaning of the study: implications for clinicians and researchers

Considerable variability occurs in the quality and completeness of morbidity coding in primary care records (Jordan *et al.*, 2004). Therefore, a review of the text records appears necessary in order to have confidence in the completeness of description of primary healthcare visits for any particular morbid disease.

The approach described here, of clinicians independently reviewing records that are mostly non-coded, and identifying the abbreviations, words, and phrases that can be used to create a software algorithm, can be applied to other illnesses which result in primary care presentations.

This approach is applicable only to relatively simple diagnoses. Its use for conditions which have very specific and multi-component case definitions, for example acute rheumatic fever or Kawasaki disease, is likely to be limited because of the inconsistencies in documentation of multiple positive or negative findings between different clinicians and by the same clinicians over time who experience competing and varied time pressures.

In summary, we describe the development of a software algorithm to screen primary care records for OM visits, and the utilization of this algorithm to increase the efficiency of identification of OM visits via clinician review of the text record. The process comprised of four steps: clinician review of the visit records, development of the algorithm, application of the algorithm to identify the healthcare visits requiring clinician review, and clinician review of records identified using the algorithm. These four steps are all required to generate precise data when using larger volumes of healthcare visits to describe presentations for specific health conditions. By reducing the number of primary care records visits requiring clinician review by 88%, the algorithm enables large volumes of free text primary care record to be reviewed by relatively small teams of clinician supported by modest budgets.

## Supporting information

Grant et al. supplementary material Grant et al. supplementary material

## Supplementary material

The supplementary material for this article can be found at https://doi.org/10.1017/S1463423625100674.

## Credit statement

Cameron Grant: Conceptualization, methodology, formal analysis, investigation, resources, data curation, writing – original draft, visualization, supervision, project administration, funding acquisition.

Marisa van Arragon: Methodology, investigation, resources, data curation, writing – review & editing, visualization, supervision.

Ellen Waymouth: Validation, investigation, resources, data curation, writing – review & editing.

Alicia Stanley: Methodology, validation, resources, data curation, writing – review & editing.

Mapui Tangi: Validation, investigation, resources, data curation, writing – review & editing.

Eamon Ellwood: Software, investigation, resources, data curation, writing – review & editing.

Carol Chelimo: Conceptualization, methodology, formal analysis, writing – review & editing, visualization, funding acquisition.
