# Supplementary material for: The identification of primary care consultation visits for otitis media: development of a software algorithm screening tool
Source: Prim Health Care Res Dev. 2026 Jan 5;27:e7. doi: 10.1017/S1463423625100674 (PMC12780419; doi:10.1017/S1463423625100674)
Supplement: Grant et al. supplementary material [file S1463423625100674sup001.docx]

**Data analysis application to identify primary care visits where otitis media was identified as a potential reason for the visit.**

**Programming Language**

A web style application using PHP: Hypertext processer^1^ (Php) coding language was developed. Php was chosen as it was the simplest coding language available to develop the programme, and has a number of pre-developed electronic file reading and text searching functions and libraries. An Apache,^2^ web server was installed on the development computer to host and run the application. As web applications are designed to process quickly, while the required application would be required to process a large number of files (4426), it was necessary to modify the Apache settings to increase the maximum processing timeout limit from the default 30 seconds to 30 minutes.

**File Types**

Files were received from the General Practices in a number of formats. They were named by the participant study ID number or National Health Index number. These were typically MS word documents, Standard word processor documents (Rich Text Files - .rtf), pdf files, or hard copy (printed/photocopied pages). These hard copies were scanned to pdf files and made computer readable using the pdf OCR text recognition function. As MS Word documents include a large amount of metadata that Php would scan as well, these files were converted to .rtf documents to strip out the metadata. To enable the Php programme to decode, scan and search pdf files, pdfparser,^3^ a freely available Php code library, was used.

**Programme operation**

The Php application scanned each file and identified visits by identifying dates. All text in between each date identified was considered a visit for the initial date. Each visit thus identified was then scanned and compared to a list of key words. To reduce false positives (e.g.: keyword “Tms”; “Tms Inflamed” = positive, “Tms normal” = false positive), each keyword identified was then compared to a list of exclusion terms (e.g. “normal”). If a key word was identified and no exclusion terms were identified, then the visit was considered positive for Otitis Media.

These key words and exclusion terms were developed in conjunction with the pilot study conducted by the physician and research nurses. The 200 child records used in the pilot study were scanned by the application and compared to the results achieved in the pilot study. Keywords and exclusion terms were added or modified as required until the closest possible match with the pilot study results was achieved.

The programme then outputted a table of positive visits with the General Practice name, the file name, the date of the visit, the key words that were matched and the visit text. If there were no positive visits for a whole patient file, the program would output “False”. This data table was then moved to a MS Access database containing the Growing Up in New Zealand General Practice and basic demographic data to allow the clinicians nurses to analyse the data.

**Pdf Issues**There were a number of different patient management systems used by the General Practices and these outputted the data in different formats. Two of these formats proved problematic for the Php application. The data in these files was presented in a columnar format, which meant that the application was unable to determine the date of the visits as all the dates were in the first column and therefore read first. The visit texts were in the second column and read next and so were considered by the programme to be one visit per page. These files were identified and scanned separately into their own output table. The Research nurses then reviewed this table manually, corrected the dates and removed the visit text that was not relevant. This table was then merged with the main data table.

**Duplicate visits**Some of the children moved between General Practices, resulting in multiple files being received for these children. As each General Practice added the previous General Practices notes to their files, this resulted in multiple duplicate visits for these children. These visits (n=530) were identified and removed from the dataset. In many cases this resulted in an entire file (n=98) being removed from the data set – usually the initial General Practice as their visits were included in subsequent General Practice data.

**Age Limit**The consent allowed for data to be collected up to seven years of age, however, this study was only concerned with visits up to four years of age. The data was compared to the Growing Up in New Zealand date of birth for each participant and visits later than four years were removed.

**References**

1. PHP: Hypertext Preprocessor. <https://www.php.net/>

2. Apache – HTTP Server Project. <https://httpd.apache.org/>

3. PDF Parser - PHP library to parse PDF files and extract elements like text. <https://www.pdfparser.org/>
